# The Role of Hyperbaric Oxygen Therapy in Neuroregeneration and Neuroprotection: A Review

**DOI:** 10.7759/cureus.62067

**Published:** 2024-06-10

**Authors:** Pedro Barata, Oscar Camacho, Clara G Lima, Ana Claudia Pereira

**Affiliations:** 1 Pathology and Laboratory Medicine, Centro Hospitalar Universitário do Porto, Porto, PRT; 2 CECLIN (Center for Clinical Studies), Hospital-Escola da Universidade Fernando Pessoa (HE-UFP), Porto, PRT; 3 Hyperbaric Medicine Unit, Unidade Local de Saúde de Matosinhos, Matosinhos, PRT; 4 Anesthesiology, Hospital Pedro Hispano, Matosinhos, PRT; 5 Faculty of Health Sciences, Universidade Fernando Pessoa (UFP), Porto, PRT

**Keywords:** neurodegenerative, oxygen, neuromodulation, hyperbaric oxygen therapy, neurogenesis

## Abstract

Neurogenesis is a high energy-demanding process, which is why blood vessels are an active part of the neurogenic niche since they allow the much-needed oxygenation of progenitor cells. In this regard, although neglected for a long time, the “oxygen niche” should be considered an important intervenient in adult neurogenesis.

One possible hypothesis for the failure of numerous neuroprotective trials is that they relied on compounds that target a highly specific neuroprotective pathway. This approach may be too limited, given the complexity of the processes that lead to cell death. Therefore, research should adopt a more multifactorial approach. Among the limited range of agents with multimodal neuromodulatory capabilities, hyperbaric oxygen therapy has demonstrated effectiveness in reducing secondary brain damage in various brain injury models. This therapy functions not only as a neuroprotective mechanism but also as a powerful neuroregenerative mechanism.

## Introduction and background

Hyperbaric oxygen therapy (HBOT) involves the therapeutic use of oxygen at higher concentrations and pressures than atmospheric levels [1,2]. HBOT is a medical treatment that uses pure oxygen in a pressurized chamber to increase the amount of oxygen in the blood, enhancing tissue oxygenation and promoting healing [1,2]. The Committee on Hyperbaric Medicine defines HBOT as “A mode of medical treatment in which the patient is entirely enclosed in a pressure chamber and breathes 100% oxygen at a pressure greater than 1 atmosphere absolute (ATA)” [3]. Even though given particular interest relatively recently, HBOT can be traced back to the 1600s. The main pioneer was a British clergyman called Henshaw, who constructed a structure called the domicilium [4]. It consisted of a pressurized chamber that was used to treat several diseases. This idea was continued by French surgeon Fontaine, who developed a pressurized mobile operating room in 1879, and later by Dr. Orville Cunningham, anesthesiologist, who created a hospital capable of functioning with three atmospheres of pressure, also known as the "Steel Ball Hospital" [4]. Due to a lack of scientific evidence that these conditions provided a therapeutic outcome, the hospital was later closed. However, the military continued the works of these scientists by demonstrating the applicability of pressurized oxygen in the treatment of diving-related injuries on navy divers [5]. Today, it has thoroughly reported evidence of its advantages in the treatment of several illnesses such as acute traumatic wounds, crush injuries, burns, gas gangrene, compartment syndrome, and non-healing ulcers [4].

Physiologically, HBOT leads to the saturation of hemoglobin with oxygen and increases the presence of unbound oxygen in the plasma. The majority of oxygen transported in the blood is bound to hemoglobin, which is typically 96%-98% saturated at standard pressure [6]. The remainder portion can be carried unbound in solution in constant balance with the bound portion. However, free oxygen can be increased, with clinical significance, under hyperbaric conditions when hemoglobin is completely saturated. Under standard pressure and breathing air conditions, free oxygen in the blood is around 0.32 mL/dL; however, the administration of 100% oxygen at normobaric conditions (1 atm) increases oxygen concentration in the blood to 2.09 mL/dL and at 3 atm this value increases to around 6.8 mL/dL (Table 1) [5]. Since tissues at rest normally extract between 5 and 6 mL of oxygen per deciliter of blood, assuming normal perfusion, the oxygen concentration in hyperbaric oxygen conditions is sufficient to satisfy tissue oxygen demand without hemoglobin contribution. Once oxygen is in solution, it is capable of improving tissue oxygenation where red blood cells cannot reach and in the case of impaired hemoglobin function or concentration [4].

**Table 1:** Effect of pressure on O2 plasma concentration. Reference [5].

Total pressure (atm)	Oxygen dissolved in plasma (% vol)	
	Breathing air	100% oxygen
1.0	0.32	2.09
1.5	0.61	3.26
2.0	0.81	4.44
2.5	1.06	5.62
3.0	1.31	6.80

The calculations for these phenomena are based on the ideal gas laws, particularly Henry's law, which states that the amount of gas dissolved in a liquid is proportional to the partial pressure of the gas above the liquid's surface. Increasing the atmospheric pressure consequently enhances the solubility of the gas. The application of Boyle’s law is also seen in many aspects of HBOT, since it can justify the decrease in air bubble size in embolic phenomena, as with increased pressure, gas volume decreases [7]. Additionally, Charles’ law is also useful for predicting temperature increases or decreases in the hyperbaric chamber and the organism [7].

The neurogenic potential refers to the capacity of neural stem cells (NSCs) or neural precursor cells (NPCs) to differentiate into mature central nervous system (CNS) cells under certain conditions [8,9], and functionally integrate into the surrounding neuronal network [8,10]. They are mostly present in specific neurogenic sites, the subventricular zone (SVZ) as well as in the subgranular zone (SGZ) in the hippocampus [11,12]. Contrary to initial beliefs, neurogenesis is a continuous process in these brain regions, and numerous experimental studies have shown that it can be altered in various neurodegenerative and neuropsychiatric diseases [13,14]. This knowledge has revealed new possibilities for treating neurodegenerative disorders that were previously considered incurable. Current therapies slow the degenerative process but still leave the brain with irreversible damage and result in little to no functional recovery. By stimulating the development of neurons, we might not only halt the progression of a disease but also reverse its harmful effects, with a high potential to restore the normal function of the brain [1,15].

Neurons present the highest energy demands, requiring a continuous supply of oxygen [16], which has generated significant interest in exploring the applicability of HBOT for neurodegenerative disorders’ treatment and prevention [17-20]. Although much remains to be discovered about its underlying mechanisms of action, HBOT's potential as a neuroprotective and neurogenic agent has been linked to the stimulation of various signaling pathways, as well as transcription factors, including hypoxia-inducible factors (HIF), Wnt, and cAMP response element-binding (CREB).

## Review

Role of oxygen in cerebral function

Oxygen (O2), as a vital substrate for energy production and cellular metabolism, is crucial for growth and development. Interestingly, normal oxygen levels present in the tissues are invariably much lower than the 156 mmHg partial pressure of oxygen (PaO2) from the air we breathe [21]: physiological concentrations range between 7 and 35 mmHg O2 [22]. This results in a “physiological hypoxia” often referred to as “in situ normoxia” [22]. The O2 supply is finely regulated in vivo. Indeed, the brain, which is the heaviest oxygen-consuming organ in the human body, depending on the region, has very low oxygen levels. The approximate levels are 15 ± 3 mmHg in the corpus callous, 20 ± 3 mmHg in the hippocampus, 27 ± 6 mmHg in the cerebral cortex, and 32 ± 4 mmHg in the thalamus, as observed in rats under isoflurane-anesthesia [22]. These measurements suggest that a steadily decreasing O2 gradient is formed as blood reaches the brain tissues [21].

O2 has been demonstrated to be essential in regulating death and differentiation of CNS in several cell lineages and is a source of cytotoxic reactive oxygen species (ROS) [21] through mitochondrial energy metabolism, which includes oxygen ions, such as superoxide O2-, the primary ROS, as well as hydroxyl radicals and peroxides, both organic and inorganic. Oxidative stress is therefore responsible for various deleterious mechanisms that promote apoptosis of CNS derivatives [23,24]. In excess, ROS are capable of promoting membrane damage, structural and functional alterations of proteins, lipids denaturation, and structural damage to DNA [25]. The brain, as the major organ in oxygen metabolization, is especially vulnerable to these deleterious effects [26]. Additionally, the brain contains a large amount of polyunsaturated fatty acids susceptible to peroxidation, which, along with elevated iron levels, act as prooxidants [27]. These may lead to the production of toxic compounds such as dienals and aldehydes (e.g., 4-hydroxynonenal), which can induce autophagic cell death or apoptosis [28]. The rapid depletion of intracellular energy through the activation of DNA repair enzymes in oxidative stress can also lead to endonuclease-mediated DNA fragmentation [29]. An increasing wealth of evidence suggests a close link between high levels of ROS in the brain and neuronal death in various neurological diseases, which include chronic neurodegenerative diseases (Parkinson’s and Alzheimer’s diseases) [24,30], acute brain injury [31-33], or psychiatric disorders (depression, autism, schizophrenia, and attention deficit hyperactivity disorder) [34]. However, in many cases, cell fate may involve a combination of effects mediated by ROS and alterations in intracellular O2 levels and hypoxia-response signaling pathways [21]. This complexity makes oxygen's role in brain function a highly complex process. In the brain, ROS are generated by microglia and astrocytes and are able to positively modulate synaptic and nonsynaptic communication between neural cells [26]. They are also capable of increasing neuronal activity by altering the myelin basic protein and by inducing synaptic long-term potentiation, which promotes synaptic plasticity and memory consolidation [26,35]. All these factors emphasize the perspective that multiple intracellular pathways contribute to the cells' determination to differentiate or undergo cell death in response to oxygen levels, which suggests that the modulation of neuroprotection and neuroregeneration in response to oxygen is also closely linked to the prooxidant/antioxidant balance in the brain.

Hypothetical mechanisms of action of HBOT

**Figure 1:**
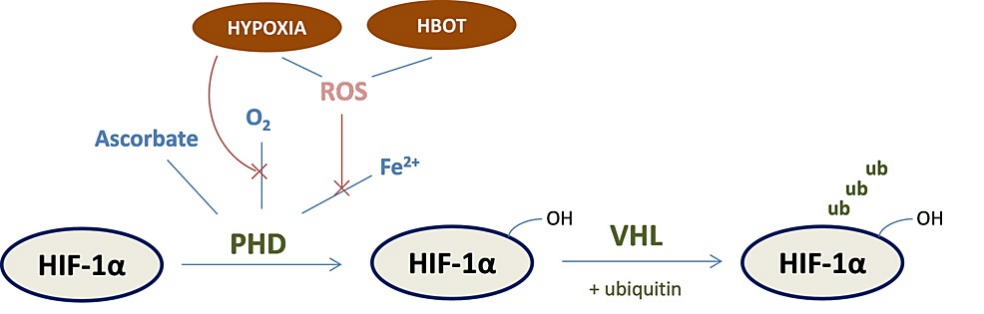
Molecular signaling pathways that lead to hypoxia-inducible factor 1 alpha (HIF-1α) stabilization or degradation. In the presence of oxygen, prolyl hydroxylase's (PHD) activity is potentiated, which leads to HIF-1α degradation through the PHD/von Hippel-Lindau protein (VHL) and ubiquitin metabolizing pathways. Hypoxic conditions inhibit PHD’s activity, therefore increasing HIF-1α stabilization and accumulation. Hyperoxia conditions also reduce PHD’s activity by oxidizing PHD-bound iron [41-47]. ROS: reactive oxygen species; ub: ubiquitin; HBOT: hyperbaric oxygen therapy; OH: hydroxide. The figure is the original work of the authors.

The molecular mechanisms underlying HBOT's promotion of cellular proliferation and differentiation are not fully elucidated; however, several signaling pathways have been suggested. The most well-studied and possibly one of the most important yet complex mechanisms is the hypoxia-inducible factor (HIF), which has a key role in cell survival or cell death [36,37]. HIF-1, an oxygen-sensitive transcriptional factor, is a heterodimer composed of two subunits, hypoxia-inducible factor 1 alpha (HIF-1α) and hypoxia-inducible factor 1 beta (HIF-1β). While the latter does not respond to altered oxygen levels, under normoxic conditions, HIF-1α is degraded rapidly through the prolyl hydroxylase (PHD)/von Hippel-Lindau protein (VHL) pathway [38]. The pVHL (von Hippel-Lindau tumor suppressor protein) is responsible for HIF-1α recognition and ubiquitination [39]. For optimal function, PHD requires oxygen, iron, 2-oxoglutarate, and ascorbic acid [40]. Hypoxic conditions inhibit the prolyl hydroxylation of HIF-1α by limiting oxygen levels available for this reaction. Therefore, the activity of the HIF-1 transcription factor is potentiated by oxygen deprivation but also by the presence of other agents that can prevent HIF-1α degradation and therefore promote its stabilization (Figure 1). During hypoxia, HIF-1 translocates from the cytoplasm to the nucleus where it binds to DNA resulting in the transcription of several genes involved in glucose homeostasis, erythropoiesis, angiogenesis, etc. [41-43]. Two of the resulting molecules, i.e., erythropoietin (EPO) and vascular endothelial growth factor, are widely known for their neuroprotective and neuroregenerative properties [43]. The mitogen-activated protein kinase (MAPK) pathway is also implicated as a regulator of HIF-1α expression [44-46]. p38 MAPK, which can be activated under hypoxia in various cell types [46,47], induces HIF-1α’s phosphorylation and shift, therefore promoting its transcriptional activity [44].

While typically reduced oxygen conditions are known to limit cell and tissue survival, these mechanisms might suggest that brain cells are able to perform better under hypoxic conditions. HIF-1 production can therefore be seen as an induced protective mechanism to diminish the deleterious effects of hypoxic events in the brain, which explains the fact that its expression is stimulated under low oxygen levels and suppressed under normoxic conditions. On the other hand, pure hyperbaric oxygen, which results in a hyperoxic environment, has been shown to promote HIF-1α stabilization via ROS production, which can oxidize PHD-bound iron [48,49]. This hypothesis is based on the model that HIF-1α is stabilized due to alterations in oxygen metabolism rather than depending solely on oxygen concentration. Furthermore, superior amounts of HIF-1 also lead to cell death through the activation of p53 [50] and BNIP3 [51]. In one report, HIF-1α is stabilized at or below 5-8% O2 but p53 accumulates below 0.2% O2 [52]. In this regard, HBOT could exert its neuroprotective effects by inhibiting the accumulation of HIF-1, and therefore stabilizing its levels [53].

**Figure 2:**
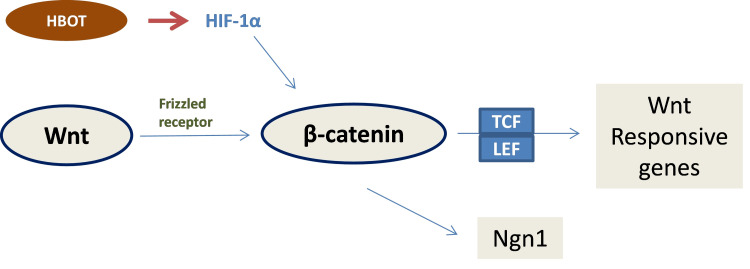
Molecular signaling pathways that lead to activation of wingless/integrated (Wnt)-responsive genes. Wnt receptor activation modulates β-catenin’s cytoplasmic translocation leading to the expression of wnt-responsive genes and Ngn1, which leads to NSC proliferation and differentiation. Under hyperoxia conditions, increased HIF-1α promotes a superior β-catenin activation [53-57]. LEF/TCF: lymphoid enhancer-binding factor-1/T-cell factor-1; HBOT: hyperbaric oxygen therapy; HIF-1α: hypoxia-inducible factor 1 alpha; Ngn1: neurogenin 1; NSC: neural stem cells. The figure is the original work of the authors.

The Wnt/β-catenin signaling pathway regulates proliferation, fate specification, and differentiation in numerous developmental stages and adult tissue homeostasis. This extremely complex signaling pathway is defined by the stabilization of cytoplasmic β-catenin upon receptor engagement by Wnt, β-catenin nuclear translocation, β-catenin interaction with LEF/TCF (lymphoid enhancer-binding factor-1/T-cell factor-1) transcription factors, and stimulation of target Wnt responsive genes [54]. This regulation extends to neural stem cells, but when cells are differentiated, this regulation is no longer observed. The β-catenin/TCF complex also appears to directly regulate the promoter of neurogenin 1 (Ngn1), a gene implicated in cortical neuronal differentiation [55]. Ngn1 promotes the differentiation of neural stem cells into neurons [56]. In vivo, the Wnt/β-catenin activity is tightly correlated with hypoxic regions in the subgranular zone of the hippocampus, which is an extremely relevant neurogenic niche [57]. HIF-1α has been shown to modulate Wnt/βcatenin signaling in hypoxic conditions by enhancing β-catenin activation and subsequent expression of its downstream effectors LEF-1 and TCF-1, which leads to NSC proliferation, differentiation, and neuronal maturation (Figure 2). It has been established that HIF-1 α is stabilized in hyperoxic conditions. Thus, the availability of oxygen may directly influence stem cell regulation by modulating the Wnt/β-catenin signaling pathway through HIF-1α [56].

**Figure 3:**
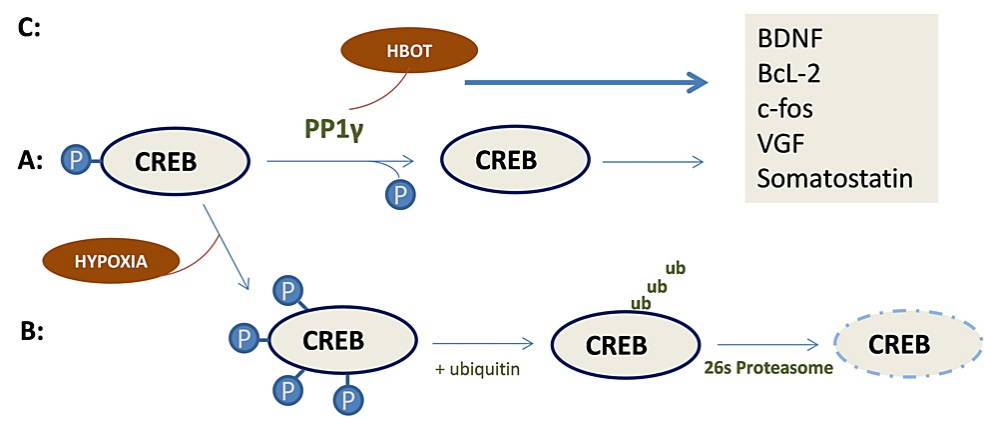
Molecular signaling pathways that lead to cAMP response element-binding (CREB) protein activation or degradation. A: During normoxia, PP1γ dephosphorylates CREB, which leads to the expression of its downstream genes (brain-derived neurotrophic factor (BDNF), B-cell lymphoma 2 (Bcl-2), protein c-FOS (c-fos), vascular endothelial growth factor (VGF), and somatostatin). B: In hypoxic conditions, the activity of isoform of the catalytic subunit of a Ser/Thr phosphatase PP1 (PP1γ) is suppressed, causing over-phosphorylation of CREB and its consequent ubiquitination and degradation by 26s proteasome. C: In hyperoxia conditions, over-phosphorylation of CREB is reversed by reactivation of PP1γ due to the presence of high oxygen levels, which potentiates the expression of its downstream genes [59,60]. PP1γ: protein phosphatase 1 gamma; ub: ubiquitin; HBOT: hyperbaric oxygen therapy. The figure is the original work of the authors.

The cAMP response element-binding (CREB) protein has been implicated in neuronal plasticity and repair, and in the formation of long-term memory via activation of its downstream genes, i.e., brain-derived neurotrophic factor (BDNF), B-cell lymphoma 2 (Bcl-2), protein c-FOS (c-fos), vascular endothelial growth factor (VGF) [58]. This activation occurs when CREB is phosphorylated, while PP1 (protein phosphatase-1) catalyzes the CREB’s dephosphorylation of PP1γ (protein phosphatase-1 gamma), which is the core subunit of this protein [59]. In hypoxic conditions, PP1γ’s activity is suppressed, which leads to the overphosphorylation of CREB, followed by its ubiquitination and degradation by 26s proteasome. When subjected to HBOT, there is a reactivation of PP1γ, which reverses CREB over-phosphorylation [60]. In addition, superior levels of oxygen also appear to directly inhibit CREB’s degradation (Figure 3) [59].

Clinical applications in neurology

Traumatic Disorders

The rationale behind the use of hyperbaric oxygen in traumatic neurological indications is based on the finding that on the periphery of the neuronal death area is located the ischemic penumbra: peri-infarct zone. This area can be defined as the hypo-perfused tissue surrounding the ischemic core in which blood flow is too low to maintain sufficient cellular metabolic activity. This area is therefore intensely subjected to deleterious metabolic processes propagated from the core to the surrounding tissue, including excitotoxicity, spreading depression, oxidative stress, and inflammatory response, which lead to the expansion of the ischemic core and the subsequent worsening of the clinical outcome [61]. Nevertheless, this area is gaining increasing interest as a target for neurorepair and neuroprotective therapies since dormant/idling neurons can be found on its periphery. Additionally, CT scans have demonstrated that what appears as gliosis (dead neurons) may actually be viable tissue for years after the traumatic insult. In this regard, HBOT can be extremely beneficial for the reperfusion of this area while delivering high oxygen levels to reactivate these “sleeping cells” and to reduce ischemic cytotoxicity on the already existing cells under stress. Additionally, HBOT is also known to reduce edema and tissue swelling due to its vasoconstriction effect [62]. Vasoconstriction in response to HBOT is likely due to the oxygen’s role in counterbalancing nitric oxide and adenosine expression, which cause vasodilation during hypoxia [63,64]. Although this could result in an inferior oxygen uptake by surrounding tissues, sufficient amounts of oxygen are present in the bloodstream to ensure maximum oxygen intake. It also reverses the reduced flexibility of erythrocytes and reduces the adherence of white cells to capillary walls, which ensure adequate oxygen permeability and normal blood flow in the brain [3]. According to Bergo and Tyssebotn [62], in the event that prolonged HBOT might be needed, which could result in a 30% decrease in blood flow to the CNS, the addition of CO2 to the gas mixture can reverse this process.

This is the argument for the use of HBOT for the treatment of stroke and post-traumatic brain injuries [65,66], as well as other disorders that lead to cerebral hemorrhage and ischemia. Nevertheless, it should be noted that it is still contra-indicated during acute stroke.

Ischemic stroke is characterized by cerebral artery occlusion, which causes regional cerebral flow reduction or interruption. Neurons have high energy demands, a trait that renders them susceptible to reduced cerebral blood flow during a stroke. Due to the appearance of the ischemic penumbra, HBOT has shown major benefits in promoting neurogenesis [20,67,68] and in improving neurological function [69] in both animal models of ischemic stroke and in clinical trials. There are also several other documented beneficial effects of HBOT in stroke treatment, including the reduction of blood-brain barrier permeability and consequently brain edema [70], attenuation of inflammation and hydroxyl radical production [71], and decreased glutamate release [72], therefore reducing brain damage. HBOT further enhances hippocampal superoxide dismutase and preserves Na+, K+-ATPase activities [73], and decreases the activity and expression of caspase-3, reduces PARP cleavage, and abolishes DNA fragmentation [69,74], which diminishes damaged cells’ apoptosis. Additionally, HBOT might extend the time window and increase the efficiency of FDA-approved recombinant tissue-type plasminogen activator (r-tPA) thrombolysis after acute ischemic stroke [75]. While ongoing HBOT improves blood flow and oxygenation to damaged brain areas, its beneficial effects are still experienced following its interruption, since the rapid passage from hyperoxia to a relative hypoxic environment leads to HIF-1α release [76,77]. This process, called oxygen cycling, reduces stroke-related injuries.

Traumatic brain injury (TBI) can be broadly defined as either physical or chemical brain damage that leads to the disruption or loss of some of its functions. Although not directly caused by poor blow flow into the brain, with similarity to ischemia, TBI can also result in a hypoxic condition. Hypoxic neurons perform anaerobic metabolism, which results in acidosis, a drastic reduction in cellular metabolic activity, and oxidative stress due to the growing apoptotic cellular core in the injured site. As this pathological environment progresses, the neurons lose their ability to maintain ionic homeostasis, free oxygen radicals accumulate, and cell membranes are degraded [78]. HBOT can therefore be a beneficial therapeutic strategy to increase oxygen availability, reduce inflammation, and promote neuroprotection and neurogenesis, but also to reduce tissue edema that commonly occurs in TBI due to brain swelling. HBOT also accelerates neo-vascularization in damaged tissues by promoting fibroblastic activity and reduces white cell adherence to capillary walls, which makes it useful in acute brain and spinal cord injury [3]. Furthermore, Bcl-2 expression enhancement, allied with an increased intracellular oxygen bioavailability might contribute to maintaining mitochondrial integrity and therefore reduce the mitochondrial pathway of apoptosis, which preserves cells in traumatic stress [79]. It is, however, necessary to take into consideration that in patients who exhibit normally perfused and oxygenated traumatic tissue, HBOT may do more harm through the action of free oxygen radicals than the potential benefits of promoting aerobic metabolism [78]. Therefore, HBOT might not be as beneficial in certain TBI cases, and the risk/benefit ratio needs to be carefully evaluated.

HBOT has also been demonstrated as an effective method in the treatment of post-concussion syndrome [80-82], which is characterized by the development of headaches, dizziness, neuropsychiatric symptoms, and cognitive impairments in 25% of mild TBI patients [83]. The above studies showed that HBOT significantly improved the symptoms and cognitive abilities of patients suffering from post-concussion syndrome.

Neurodegenerative Disorders

Similarly to the role in stimulating neurogenesis in the ischemic penumbra, HBOT is also capable of promoting neurogenesis in other types of neurological diseases, through similar molecular signaling pathways [84-86]. Neurodegenerative disorders, such as Parkinson’s disease (PD), Alzheimer’s disease (AD), and cerebral palsy, are loosely characterized by the loss of cell function in the production and release of certain neurotransmitters as well as neuroinflammation that ultimately leads to cell loss accompanied by impaired motor or cognitive functions.

PD is a progressive neurodegenerative condition marked by symptoms such as resting tremor, rigidity, and bradykinesia [87]. Neuroinflammation and oxidative stress occur as a consequence of exacerbated ROS production in PD individuals [88]. Elevated production of ROS and an imbalance in antioxidant defense and repair mechanisms result in the loss or apoptosis of dopaminergic neurons in the substantia nigra [89]. Thus, the reversion of cellular oxidative damage through HBOT might represent an effective strategy for PD treatment, since increasing evidence suggests that superoxide dismutase, glutathione peroxidase, and catalase activity are increased after repeated HBOT treatments [90,91]. Additionally, HBOT also protects against Bax/Bcl-2-mediated apoptosis and alleviates the production of glial fibrillary acidic protein (GFAP) in the substantia nigra, which results in protection against loss of neurons in PD [87].

AD is a progressive neurodegenerative disorder that leads to cognitive impairment and progressive dementia [92]. It is characterized by the accumulation and deposition of Aβ (amyloid β) peptides in the brain, which leads to Aβ-induced free radical-mediated damage, neurofibrillary tangles, and neuronal cell death [93]. Recent evidence suggests that oxidative stress and damage in mitochondrial function also play an important role in the development of AD [94]. Therefore, by increasing the activity of superoxide dismutase and glutathione peroxidase [79] and reducing the activation of the mitochondrial apoptosis pathway [95], HBOT can significantly benefit AD symptom management. It is suggested that HBOT may reduce cell toxicity and oxidative stress by blocking mitochondria-mediated apoptosis signaling in AD [96].

Cerebral palsy covers a group of non-progressive, but often changing, motor impairment syndromes secondary to lesions or anomalies of the brain in the early stages of development. Results on the effectiveness of HBOT in cerebral palsy treatment are very controversial. Some studies report improved gross motor function, improved fine motor function, reduced muscle spasticity [97], and improvements in speech, attention, memory, and functional skills [98,99]. However, Collet et al. (2001) [98] reported that no significant difference was observed between 1.5 atm 100% oxygen and room air with slightly elevated pressure, and Rossignol and team [99] reported unaffected intracellular oxidative stress markers in HBOT regimens. There is, therefore, an increasing need to further study the mechanisms underlying HBOT’s role in cerebral palsy to clarify ongoing conflict in this field.

## Conclusions

Clear evidence has so far demonstrated HBOT’s potential role in neuroprotection and neuroregeneration through several molecular and physiological signaling pathways. The applicability of these findings has been intensely linked with the treatment of neurological disorders with a traumatic background. For neurodegenerative and neuropsychiatric disorders, a positive trend can be seen in the interest of using HBOT, which reflects its growing potential.

While HBOT offers potential benefits, it is essential to consider its limitations, associated risks, and the need for individualized care. The increased pressure during HBOT sessions can pose a risk of barotrauma. Moreover, prolonged exposure to high levels of oxygen at elevated pressures can lead to oxygen toxicity. This potentiates the risk of seizures and pulmonary issues, among other complications. Another important aspect is regarding patients who are already under specific treatments or present previous medical conditions for which HBOT may be contraindicated or require further analysis due to potential interaction with other pharmaceutical therapies. Nevertheless, these are limitations that, under proper analysis, can be adjusted to the patient’s needs in a personalized medicine manner. For that, strictly controlled clinical trials need to be created to speed the process of HBOT’s acceptance as a possibly effective treatment. This would enable HBOT’s establishment as a preventive and/or therapeutic approach for neurological disorders.
